# Supplementary material for: Nanoscale reshaping of resonant dielectric microstructures by light-driven explosions
Source: Nat Commun. 2023 Oct 21;14:6688. doi: 10.1038/s41467-023-42263-w (PMC10590427; doi:10.1038/s41467-023-42263-w)
Supplement: Supplementary file 1 — Supplementary Information [file 41467_2023_42263_MOESM1_ESM.pdf]

Nanoscale reshaping of resonant dielectric microstructures by light-driven explosions. Supplementary Information

Maxim R. Shcherbakov,[1,2,*] Giovanni Satrorello,[3,4] Simin Zhang,[5] Joshua Bocanegra,[1,6] Melissa Bosch,[3,7] Michael Tripepi,[8,9] Noah Talisa,[10,9] Abdallah AlShafey,[9] Joseph Smith,[11] Stephen Londo,[12] François Légaré,[12] Enam Chowdhury,[5,9,13] Gennady Shvets[3]

[1]*Department of Electrical Engineering and Computer Science, University of California, Irvine, CA 92697, USA*
[2]*Beckman Laser Institute and Medical Clinic, University of California, Irvine, CA 92612, USA*
[3]*School of Applied and Engineering Physics, Cornell University, Ithaca, NY 14850, USA*
[4]*Cornell NanoScale Science and Technology Facility, Cornell University, Ithaca, NY 14853, USA*
[5]*Department of Material Science and Engineering, The Ohio State University, Columbus, OH 43210, USA*
[6]*Department of Physics, University of California, Irvine, CA 92697, USA*
[7]*Department of Physics, Cornell University, Ithaca, NY 14850, USA*
[8]*Physics Department, Hillsdale College, Hillsdale, MI 49242, USA*
[9]*Department of Physics, The Ohio State University, Columbus, OH 43210, USA*
[10]*Johns Hopkins University Applied Physics Laboratory, Laurel, MD 20723, USA*
[11]*Physics Department, Marietta College, Marietta, OH 45750, USA*
[12]*INRS-EMT, Québec J3X 1P7, Canada*
[13]*Department of Electrical and Computer Engineering, The Ohio State University, Columbus, OH 43210, USA*

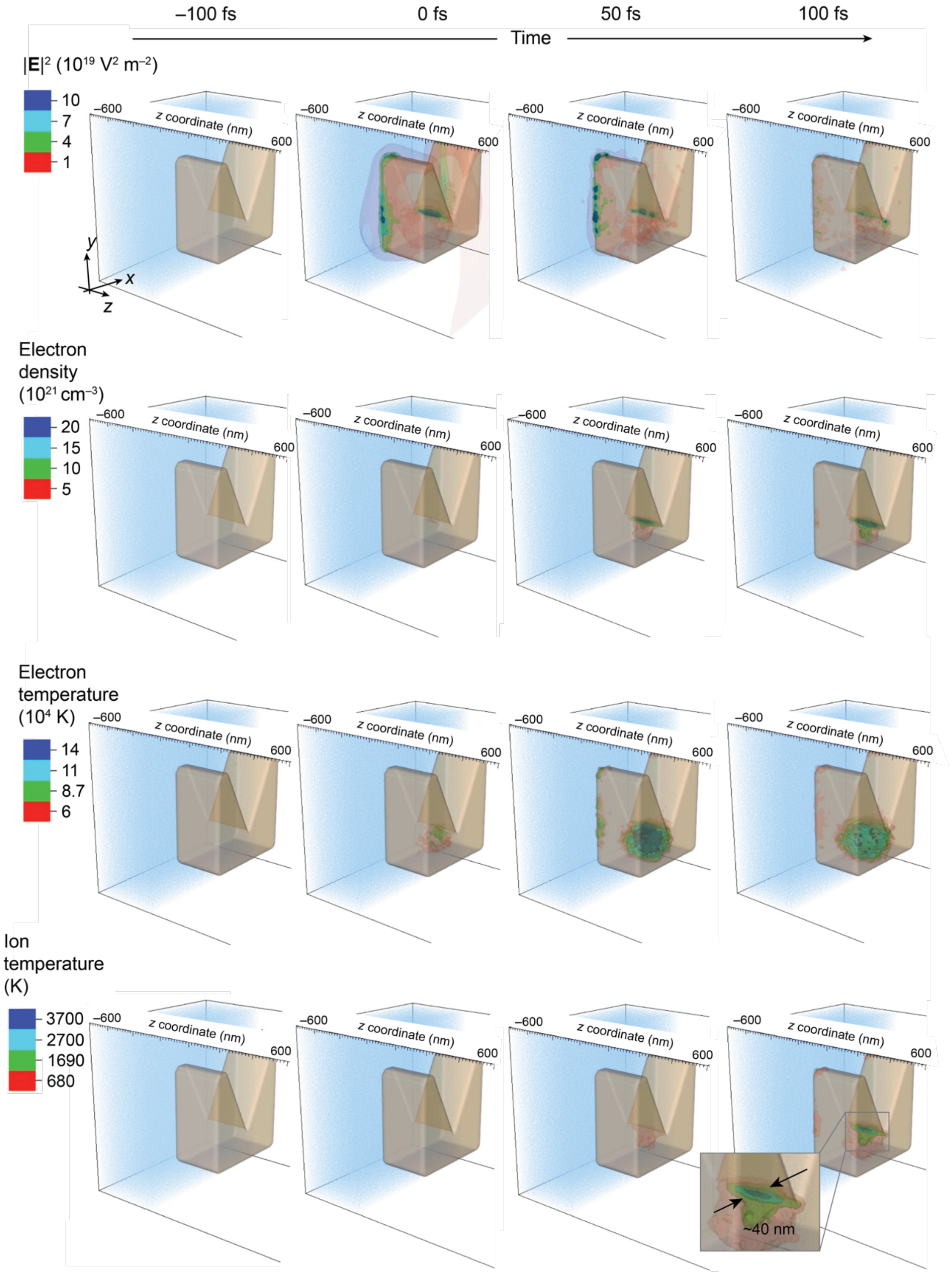

Supplementary Figure 1. PIC results for a scaled-down microresonator and pump wavelength. The scaling factor is 0.5, where $\lambda_L = \lambda_R = 1.95$ µm, and all the dimensions of the simulation are halved. The minimum projected feature from the final ion temperature distribution is around 40 nm.

Supplementary Note 1. The Role of Native Oxide

Since the experiments were conducted at ambient conditions, the existence of the native oxide layer on silicon was presumed. However, we render the role of the native oxide (thickness ~2 nm) negligible compared to the bulk of the microresonators with their characteristic dimensions. Since the wavelength of the resonance is, as an approximation, proportional to the $n_{\mathrm{eff}} - 1$, where n_{eff} is the effective refractive index of the resonator. The overall change of the resonator mode's central wavelength due to the native oxide can be roughly estimated as $\Delta\lambda/\lambda \approx \Delta n_{\mathrm{eff}}/(n_{\mathrm{eff}} - 1)$, where $\Delta n_{\mathrm{eff}} \approx n_{\mathrm{Si}} - n_{\mathrm{Si+SiO_2}}$, $n_{\mathrm{Si}} = 3.4$, and $n_{\mathrm{Si+SiO_2}}$ is the adjusted effective refractive index due to the addition of the native oxide, estimated at $n_{\mathrm{Si+SiO_2}} = (V_{\mathrm{Si}} n_{\mathrm{Si}} + V_{\mathrm{SiO_2}} n_{\mathrm{SiO_2}})/(V_{\mathrm{Si}} + V_{\mathrm{SiO_2}})$, where V is the respective volume of the material. At 2 nm thickness of SiO_2 and approximating the microresonator as a cuboid with $0.6\ \mu\mathrm{m} \times 1\ \mu\mathrm{m} \times 1\ \mu\mathrm{m}$ dimensions, the ratio of the volumes $\frac{V_{\mathrm{SiO_2}}}{V_{\mathrm{Si}}} \approx 0.015$, and $n_{\mathrm{SiO_2}} = 1.5$, the overall n_{eff} correction due to the oxide is $\Delta n_{\mathrm{eff}} \approx 0.03$, making the central wavelength correction $\Delta\lambda \approx 50$ nm, which is well within the bandwidths of the pulse and the resonance.

Supplementary Note 2. The Role of Resonator Heating

In our particle-in-cell calculations, we have estimated the amount of heat that is generated through the relevant processes of free carrier generation and thermalization with the lattice. Fig. 5 of the main text shows the overall lattice temperature elevation and its distribution throughout the volume of the material, peaking at around 6,000–7,000 K in the hot spot. In this dynamic picture, the elevated temperature profile will remove the material from the V groove in the microresonator. However, when averaged over the material's volume, the resonator's overall temperature rise is mild. With the hot spot volume being roughy $0.1\ \mu\mathrm{m} \times 0.1\ \mu\mathrm{m} \times 0.6\ \mu\mathrm{m} = 0.006\ \mu\mathrm{m}^3$, and the resonator volume estimated at $1\ \mu\mathrm{m} \times 1\ \mu\mathrm{m} \times 0.6\ \mu\mathrm{m} = 0.6\ \mu\mathrm{m}^3$, the averaged overall temperature rise of the resonator is estimated around $50 - 60$ K which is far below the melting point of silicon (1,683 K), without taking into account the heat dissipation in the substrate and the removal of heat by the ejected particulates. Therefore, our fine-tuning of the laser beam intensity allowed us highly localized material modification and removal without any significant modification to the remainder of the material. The studies of the microscopic changes, such as localized amorphization of Si, represent an exciting avenue for research that will be considered for our future studies.
