# Supplementary material for: Nanoscale reshaping of resonant dielectric microstructures by light-driven explosions
Source: Nat Commun. 2023 Oct 21;14:6688. doi: 10.1038/s41467-023-42263-w (PMC10590427; doi:10.1038/s41467-023-42263-w)
Supplement: Supplementary file 3 — Description of Additional Supplementary Files [file 41467_2023_42263_MOESM3_ESM.pdf]

Description of Additional Supplementary Files

Supplementary Movie 1. PIC-calculated evolution of the electric field in the M-shaped resonator.

Supplementary Movie 2. PIC-calculated evolution of the electron in the M-shaped resonator superimposed over the electric field (the brown contour corresponds to $E = 2.6$ V nm^{-1}).

Supplementary Movie 3. PIC-calculated evolution of the electron temperature in the M-shaped resonator.

Supplementary Movie 4. PIC-calculated evolution of the ion temperature in the M-shaped resonator.

Supplementary Movie 5. PIC-calculated evolution of ion density in the resonator, *x*-*y* middle section.

Supplementary Movie 6. PIC-calculated evolution of ion density in the resonator, *y*-*z* middle section.
